# The Strengthening Exercises in Shoulder Impingement trial (The SExSI-trial) investigating the effectiveness of a simple add-on shoulder strengthening exercise programme in patients with long-lasting subacromial impingement syndrome: Study protocol for a pragmatic, assessor blinded, parallel-group, randomised, controlled trial

**DOI:** 10.1186/s13063-018-2509-7

**Published:** 2018-03-02

**Authors:** Mikkel Bek Clausen, Thomas Bandholm, Michael Skovdal Rathleff, Karl Bang Christensen, Mette Kreutzfeldt Zebis, Thomas Graven-Nielsen, Per Hölmich, Kristian Thorborg

**Affiliations:** 1 Department of Physiotherapy and Occupational Therapy, Faculty of Health and Technology, Metropolitan University College, Sigurdsgade 26, DK-2200 Copenhagen N, Denmark; 2 Sports Orthopaedic Research Center – Copenhagen (SORC-C), Department of Orthopedic Surgery, Copenhagen University Hospital, Amager-Hvidovre, Kettegårds Allé 30, DK-2650 Hvidovre, Denmark; 3 Physical Medicine and Rehabilitation Research-Copenhagen (PMR-C), Department of Physical and Occupational Therapy, Copenhagen University Hospital, Amager-Hvidovre, Kettegårds Allé 30, DK-2650 Hvidovre, Denmark; 4 Clinical Research Centre (056), Copenhagen University Hospital, Amager-Hvidovre, Kettegårds Allé 30, 2650 Hvidovre, Denmark; 5 Research Unit for General Practice in Aalborg, Department of Clinical Medicine, Aalborg University, Aalborg, Denmark; 6 Department of Health Science and Technology, Faculty of Medicine, Center for Sensory-Motor Interaction (SMI), Aalborg University, Aalborg, Denmark; 7 Department of Biostatistics, University of Copenhagen, Copenhagen, Denmark; 8 Center for Neuroplasticity and Pain (CNAP), SMI, Department of Health Science and Technology, Faculty of Medicine, Aalborg University, Fredrik Bajers Vej 7, Aalborg, Denmark

**Keywords:** Shoulder, Impingement, Pragmatic, Strength, Progressive, Exercise, RCT, Rotator cuff, Adherence, Sensitisation

## Abstract

**Background:**

Subacromial impingement syndrome (SIS) is a painful, and often long lasting, shoulder condition affecting patient function and quality of life. In a recent study, we observed major strength impairments in shoulder external rotation and abduction (~30%) in a population of patients with pronounced and long-lasting SIS. However, the current rehabilitation of such strength impairments may be inadequate, with novel rehabilitation programmes including exercise therapy only improving external rotation strength by 4–13%.

As these previous studies are the basis of current practice, this suggests that the strengthening component could be inadequate in the rehabilitation of these patients, and it seems likely that more emphasis should be placed on intensifying this part of the rehabilitation.

The purpose of this study is to investigate the effectiveness of a programme consisting of progressive home-based resistance training using an elastic band, aimed at improving shoulder external rotation and abduction strength, added to usual care and initiated shortly after diagnosis has been established.

**Methods:**

A pragmatic randomised controlled superiority trial will be conducted, including 200 patients with pronounced and long-lasting SIS, diagnosed using predefined criteria. Participants will be randomised to receive either an add-on intervention of progressive home-based resistance training using an elastic band in addition to usual care or usual care alone in a 1:1 allocation ratio. The randomisation sequence is computer generated, with permuted blocks of random sizes. The primary outcome will be change in Shoulder Pain And Disability Index (SPADI) score from baseline to 16 weeks follow-up. Outcome assessors are blinded to group allocation. Intervention receivers will be kept blind to treatment allocation through minimal information about the content of the add-on intervention and control condition until group allocation is final. Analyses are performed by blinded data analysts.

**Discussion:**

If effective, the simple shoulder strengthening exercise programme investigated in this trial could easily be added to usual care. The usefulness of the trial is further supported by the magnitude of the problem, the information gained from the study and the pragmatism, patient centeredness and transparency of the trial.

**Trial registration:**

The trial is pre-registered at ClinicalTrials.gov with the ID NCT02747251 on April 19, 2016.

**Electronic supplementary material:**

The online version of this article (10.1186/s13063-018-2509-7) contains supplementary material, which is available to authorized users.

## Background

Shoulder disorders are the second most frequent musculoskeletal reason for contacting a general practitioner [1]. Nearly half of these incidents are categorised as subacromial impingement syndrome (SIS) [2], a painful, and often long lasting [3], condition affecting patient function and quality of life [4]. In Denmark, the incidence of SIS reported in primary care is approximately 8 per 1000 inhabitants per year [5]. Further, with an average yearly cost per incident case of shoulder disorder of €4000 [6], as reported in Sweden, the societal costs related to SIS are noteworthy. However, most of the costs (74%) are related to a sub-group of patients (12%) with high pain intensity and more pronounced and long-lasting disability [7]. In this sub-group, sick leave from paid work accounts for more than half (61%) of the costs [7].

The latest systematic review on the efficacy of exercise therapy in the treatment of SIS suggests that an exercise intervention improves patient-reported function and pain in patients with long-lasting SIS (≥ 3 months), to a degree equivalent to surgery followed by post-operative rehabilitation [8]. In addition, an intervention including specific exercises significantly reduced the amount of operations in patients on the waiting list for surgery for SIS [9]. Consistent with these studies, the Danish national clinical guidelines for treatment of SIS recommends an exercise intervention lasting at least 3 months [10]. However, it is unknown which specific components of the heterogeneous exercise interventions are associated with a better outcome [11] and, though not demonstrated in patients with long-lasting SIS, patient adherence to the prescribed exercise interventions is also likely to moderate the effects of such interventions.

### Mechanisms

Resistance training, aimed at strengthening the rotator cuff muscles and scapula stabilising muscles, is an important component of most novel exercise interventions for long-lasting SIS [9, 12, 13]. This seems relevant as patients with SIS have significant force impairments in both the glenohumeral and the scapulothoracic joint [4, 14, 15]. However, these force impairments are most pronounced in the glenohumeral joint, with a 33% deficit in external rotation force and a 29% deficit in abduction force compared to only 8% and 18% force deficit in protraction and retraction of the scapula, respectively [15]. This suggests that specific training of the glenohumeral muscles is especially relevant. Such specific resistance training of the, often degenerated [16], rotator cuff muscles and tendons also seems relevant as this training modality is known to improve muscle and tendon health through various pathways [17].

### Need for a trial

It is uncertain if the novel exercise intervention programmes, aimed at patients with long-lasting SIS, are focusing sufficiently on strengthening exercises. Accordingly, changes in muscle force are only tested in a few randomised controlled trials (RCTs), including resistance training in the rehabilitation of patients with SIS [12, 13, 18, 19]. In these studies, maximum force in external rotation increased by only 4–15% in average from baseline to follow-up in patients doing strengthening exercises [12, 13, 18, 19], which is far from restoring the 33% impairment in external rotation force previously reported [15]. Though strength gain may not be the primary aim of the intervention, the sparse effect on this objective outcome points towards a possible gap in the rehabilitation of patients with long-lasting SIS. This suggests that the exercise dose received by patients might have been too small, either because the prescribed resistance training programmes were too mild, or simply due to lack of adherence to the prescribed programmes. The trial described in this protocol will aim to investigate the effectiveness of adding a simple shoulder strengthening exercise programme to usual care in patients with long-lasting SIS.

### Existing knowledge

To further ensure that we do not initiate a redundant trial, we have performed a systematic literature review. On January 26, 2016, we performed a systematic literature search on controlled trials investigating the effect of resistance training in patients with SIS. We searched Medline via PubMed (86 hits) and Embase via OVID (38 hits) using search strings for condition (shoulder impingement) and intervention (resistance training). We also searched www.clinicaltrials.gov for registered (finalised, ongoing or planned) trials. We only identified one trial [18] in which the effect of resistance training was isolated as the active part of the intervention. Additionally, in a recent systematic review, only one study [20] investigating the effect of an exercise intervention in patients with persistent SIS was identified, and in that study the exercise intervention was compared to surgery. In conclusion, the existing evidence regarding the effect of resistance training in patients with persistent SIS is very limited and we therefore consider this to provide a clear-cut ethical, scientific and economic justification for the trial described in this protocol.

### Dose selection

As previously stated, the exercise dose received by patients might have been too small in previous studies, either because the prescribed resistance training programmes were too mild or simply due to lack of adherence to the prescribed programmes. In the study by Lombardi et al. [18], patients allocated to an intervention consisting of supervised strengthening exercises only increased 9% in external rotation strength (derived from Lombardi et al. [18]), which was not significantly different from the waiting list control group. As exercise sessions were supervised, this absence of effect is likely a consequence of the prescribed dose being too small, with exercises for flexion, extension, internal and external rotation performed twice a week in 8 weeks with 2 × 8 repetitions at 50% and 70% of 6RM, respectively. One way to efficiently increase the exercise dose in rehabilitation is to include home-based exercises, as was done in a study by Bennell et al. [12]. In that study, an intervention consisting of manual therapy and home exercises was found superior to placebo in improving abduction strength (significant 15% increase), but not external rotation strength (non-significant 4% increase), pain scores or patient-reported function at the end of treatment. The limited gains in shoulder strength observed in the study by Bennell et al. [12] might also be a consequence of an insufficient exercise dose received by the patients. However, it is difficult to determine whether the sparse improvements in shoulder strength are due to the programme being too mild or because of low adherence, as the amount of external resistance applied to exercises is not described. Nevertheless, exclusion of non-adherent patients from the analyses did not alter the results, indicating that the sparse improvements in shoulder strength were not only due to low adherence.

In the current study, we aim to increase the received dose of strengthening exercises aimed at the rotator cuff muscles in patients with long-lasting SIS without aggravating their symptoms and with minimal risk of adverse events. To achieve this, the intervention will start with exercises using low relative resistance but high volume (number of sets per session and frequency of sessions). This approach is also relevant, as resistance training with low intensity/resistance, high frequency and approximately 3–4 sets per muscle group is associated with the highest gains in muscle strength in untrained individuals [21]. All sets are to be continued to failure, as this maximises the gain in local muscle endurance [22]. Furthermore, the low resistance will minimise pain during exercises and protect the muscle from overload injuries, ensuring that all patients are able to complete the programme.

The focus of exercises is on isometric holds and slow dynamic strengthening with a long time under tension (TUT). A high TUT is achieved through exercises with a low contraction velocity and the inclusion of an isometric component to increase the exercise stimulus without increasing external resistance; this in turn reduces the peak load on the involved, and possibly damaged [16], muscles and tendons. Thus, exercises become safer and less painful, which again will allow patients to actually adhere to the exercise intervention. Furthermore, the isometric part will be performed with the shoulder in approximately 30–45 degrees of scaption, the position in which the compression forces on the supraspinatus muscle and tendon are lowest [23, 24]. Exercises will mainly focus on external rotation and abduction, functions in which both supraspinatus and infraspinatus muscles are highly active [25].

### Possible effect modifiers

Patients with long-lasting SIS have lower mechanical pressure pain thresholds (PPT), both at the shoulder region and in other anatomical regions (such as the tibialis anterior), which may reflect an increased manifestation of peripheral and central pain sensitisation [26]. Pain sensitisation modulates the patients’ pain experience and, furthermore, the presence of hyperalgesia or referred pain before decompression surgery significantly worsens the outcome after 3 months [26], while more pain catastrophizing is related to persistence of symptoms in patients with long-lasting SIS [27]. It therefore seems likely that manifestations of peripheral and central pain sensitisation, and peripheral adaptations to long-lasting pain, could also affect the outcome of an exercise intervention. This could be either through an alteration in adherence to the intervention due to pain sensitisation, or through other mechanisms related to central pain sensitisation and peripheral adaptations.

The effect of scapula function on treatment outcome in patients with SIS is often debated, and one could argue that the increased emphasis on rotator cuff muscles, as is proposed in the current trial, would neglect an important aspect of the rehabilitation. Accordingly, it has been suggested that rotator cuff emphasis should only be after scapula control is achieved, as insufficient dynamic stability of the scapula could cause abnormal shoulder kinematics and impingement symptoms [28]. Assuming this, the effect of the add-on intervention in the current study, focusing mainly on strengthening exercises for the rotator cuff, could be modified be the presence of scapula dyskinesis. However, Mulligan et al. [29] recently found that treatment outcome was not significantly different between patients randomised to scapula stabilisation first, or rotator cuff strengthening first, questioning the importance of maintaining a scapula focus in the beginning of rehabilitation.

### Explanation for choice of comparators

This study aims to investigate the effectiveness of adding a simple shoulder strengthening exercise programme to usual care in patients with long-lasting SIS. Therefore, the relevant control condition is usual care, which is to be compared to the intervention condition involving an add-on exercise programme and usual care.

This is relevant because usual care for patients that are covered by the Danish National Guidelines for Treatment of SIS [10], and hence have a medically justified need for general rehabilitation, is referral to general rehabilitation under the Danish health act § 140. Such rehabilitation is likely based on the most novel evidence from previously mentioned RCTs [12, 13, 18], where only limited gains in muscle strength are found. It therefore seems unlikely that the current treatment addresses the issue of glenohumeral muscle strength to a sufficient degree.

Furthermore, direct monitoring of adherence to the add-on intervention makes it possible to conduct secondary analysis, investigating the relationship between exercise adherence and changes in both strength and patient-reported function.

## Methods

### Objectives

#### Primary research question

Based on the literature reviewed in the “Background” section, we asked the following research question:

Is a 16-week simple home-based shoulder strengthening exercise programme, in addition to usual care, superior to usual care alone for improving patient-reported shoulder function 16 weeks after baseline, measured using the Shoulder Pain And Disability Index (SPADI), in patients with persistent (> 3 months) subacromial impingement syndrome referred to further examination at a hospital?

The research question was framed using the PICOT model [30] with the following options for each element:

- **P**opulation: Patients with persistent SIS (> 3 months)
- **I**ntervention: 16-week simple home-based shoulder strengthening exercise programme added to usual care
- **C**ontrol: Usual care
- **O**utcome: Patient-reported shoulder function
- **T**ime frame: 16 weeks after baseline

#### Primary objective

The primary objective is therefore to investigate the effectiveness of adding a 16-week simple home-based shoulder strengthening exercise programme to usual care, compared to usual care alone, on changes in patient-reported shoulder function (SPADI score) from baseline to end of treatment (16 weeks) in patients with SIS.

#### Primary research hypothesis

Our hypothesis is that a 16-week simple home-based shoulder strengthening exercise programme, in addition to usual care, is superior to usual care alone for improving patient-reported shoulder function 16 weeks after baseline, measured using the SPADI, in patients with persistent (> 3 months) subacromial impingement syndrome referred to further examination at a hospital.

#### Secondary objectives

1. To investigate the effectiveness of adding a 16-week simple home-based shoulder strengthening exercise programme to usual care, compared to usual care alone, on changes in shoulder abduction and external rotation strength from baseline to end of treatment (16 weeks) in patients with SIS.
2. To investigate the modifying effects of pain sensitisation, pain catastrophizing and scapula function on the effectiveness of adding a 16-week simple home-based shoulder strengthening exercise programme to usual care, compared to usual care alone, on changes in patient-reported shoulder function and shoulder abduction and external rotation strength from baseline to end of treatment (16 weeks) in patients with SIS.
3. To investigate the dose-response relationship between objectively monitored adherence to the add-on intervention and change in patient-reported shoulder function, shoulder abduction strength and external rotation strength.
4. To investigate the relationship between central and peripheral pain sensitisation and change in patient-reported shoulder function, shoulder abduction strength and external rotation strength, and to what degree this is mediated through adherence to the intervention.

### Trial design

The SExSI-Trial is a pragmatic, assessor blinded, randomised, controlled superiority trial, with a two-group parallel design. Patients will be randomised to either usual care or a home-based intervention consisting of progressive high volume resistance training in addition to usual care with a 1:1 allocation. The primary end-point will be change in patient-reported shoulder function 16 weeks after baseline.

This clinical trial protocol is based on the PREPARE Trial guide [31] and the SPIRIT checklist [32] (Additional file 1). The trial report will adhere to the CONSORT guidelines for reporting parallel group randomised trials, the CONSORT extension for pragmatic trials http://www.consort-statement.org, and the TIDieR template for intervention description and replication [33].

### Study setting and eligibility criteria

Participants will be recruited from the secondary care orthopaedic outpatient clinic at Hvidovre Hospital. Consecutive sampling will be used to ensure generalisability of the results. Accordingly, all patients who are referred for the first time to the clinic for examination of their current shoulder disorder lasting at least 3 months, who are living in the Capitol Region of Copenhagen, Denmark, who are not pregnant, do not permanently use strong pain medication (defined as morphine analgesic or similar), are aged 18–65 and are considered able to understand spoken and written Danish will be evaluated for eligibility by an orthopaedic specialist. All eligible patients will be provided with written information about the study.

Patients will be invited to participate if they meet the following inclusion criteria: (1) At least three positive of the five diagnostic tests for subacromial impingement syndrome (Hawkins-Kennedy test, Neer’s test, pain-full arc, Resisted External Rotation test and Jobe’s test) [34]; (2) Have been provided with or offered a rehabilitation plan due to a medically justified need for general rehabilitation after discharge from the hospital under the Danish Health Act § 140; and (3) hand in a completed SPADI questionnaire on the day of the medical examination.

Patients will be excluded if they fulfil any of the exclusion criteria, namely (1) have a radiologically verified new or previous fracture related to the shoulder joint, including the scapula; (2) have radiologically verified glenohumeral osteoarthritis; (3) have a clinically suspected luxation or sub-luxation of the glenohumeral, acromioclavicular or sternoclavicular joint; or (4) have a clinically suspected labral lesion, complete traumatic tear of the rotator cuff, frozen shoulder or other competing diagnoses (i.e. rheumatoid arthritis, cancer, neurological disorders, fibromyalgia, psychiatric illness).

### Interventions

Both groups will receive usual care consisting of an offer of referral to general rehabilitation in the municipal clinic under the Danish health Act § 140, sometimes with the option to instead choose a private physiotherapy clinic, partly at their own expense. The referral is standard procedure when a patient with SIS is considered to have a medically justified need for general rehabilitation, as identified during the examination at the orthopaedic outpatient department (secondary care unit). This procedure is based on the Danish National clinical guideline on diagnostics and treatment of patients with selected shoulder disorders [10], as exercise therapy is recommended as first line of treatment for SIS.

In the context of this study, usual care includes all treatment received by a patient during the time between baseline and follow-up, except that included in ‘Strengthen your Shoulder’. Therefore, usual care might include a range of treatment modalities including advice, stretching, exercises, manual therapy, massage, acupuncture and electrotherapy at the discretion of the treating physiotherapist and doctor. A description of usual care following the TIDieR guidelines [33] is included in Additional file 2.

Patients in the intervention group will, in addition to usual care, receive instructions regarding a home-based, progressive, high volume resistance-training programme immediately after randomisation. Instructions in the exercises are provided at baseline (week 0) and after 2, 5, 10 and 16 weeks. The exercise programme has been developed in cooperation with a team of experts with extensive knowledge of both exercise physiology and rehabilitation and training of patients with shoulder disorders (MC, TB, KT and MR). The design of the exercises, and their progression, is based on the relevant literature regarding exercise physiology, shoulder biomechanics and strength training principles for both healthy people and patients with shoulder disorders. A standard protocol for individual adaptation of the programme based on pain response is part of the intervention.

A thorough description of the intervention, and modifications of the intervention, following the TIDieR guidelines [33], is included in Additional file 3, and the full set of strength training descriptors, as suggested by Toigo and Boutellier [35], are presented in Table 1. An online instruction video of the exercises will be made available upon publication of the main results of the trial, and a link to these is provided in Additional file 3.

**Table 1:** Strength training descriptors [35] of the exercises performed in the intervention group

	Phase 1	Phase 2	Phase 3
Load magnitude	15–20 RM	10–15 RM	8–10 RM
Number of repetitions	To volitional muscular fatigue	To volitional muscular fatigue	To volitional muscular fatigue
Number of sets	3 sets	4 sets (2 per exercise)	6 sets (2 per exercise)
Rest in-between sets	60 s	60 s	60 s
Sessions per week	7 per week	3.5 per week	3.5 per week
Duration of experimental period	5 weeks	5 weeks	6 weeks
Contraction modes per rep			
Concentric	2 s	2 s	2 s
Isometric	5 s	5 s	5 s
Eccentric	2 s	2 s	2 s
Rest between reps	2 s	2 s	2 s
Time under tension	9 s	9 s	9 s
Volitional muscular fatigue	Yes	Yes	Yes
Rest between sessions	24 h	48 h	48 h
Anatomical definition of exercise	Yes	Yes	Yes
Range of movement	Exercise 1: 80° ER	Exercise 1: 80° ER Exercise 2: 45° ABD	Exercise 1: 80° ER Exercise 2: 45° ABD Exercise 3: 45° ER

#### Interventions – adherence

Lack of adherence to an exercise intervention is a major problem when aiming at investigating the effect of an intervention. In the previously described studies that include unsupervised resistance training in rehabilitation of SIS patients, monitoring adherence is either done using a log-book filled in by the patients [9, 12] or not described at all [13]. Using only patient-reported measures of adherence may limit the possibility of detecting which exercise dose is actually received by the patient. Accordingly, in a recent systematic review, the authors did not identify a single measure of patient-reported adherence to unsupervised home-based rehabilitation exercises to be sufficiently investigated [36]. Furthermore, findings from one previous study revealed that the dose of exercises reported in exercise diaries were 2.3 times higher than that collected through a system which monitored the exact TUT, being the total time a muscle resists weight during each set [37]. This clearly underlines the relevance of collecting objective data on exercise adherence. In addition, it seems unlikely that patients are always able to ‘take’ precisely the exercise dose prescribed. This issue was unfolded in a recent study, revealing that, just 2 weeks after initial instructions, only 7 out of 29 young healthy individuals were able to use the correct TUT, when performing shoulder exercises with an elastic band [38].

In the SExSI-Trial, adherence to the add-on progressive high volume resistance training intervention will be captured using the BandCizer^©^. The BandCizer^©^ is a small device which is mounted on the elastic band during exercises to measure the TUT, number of repetitions and total work load for all exercises performed. Exactly TUT is a promising objective measure of exercise adherence, for which the BandCizer^©^ is specifically developed and validated [39–41]. By directly monitoring the actual TUT for the prescribed exercises a more precise and objective distinction between adherent and less adherent patients will be possible.

In both the intervention group and the control group, the time spent on exercises related to usual care will be monitored by self-report, using a text-message based system (SMS-track^©^). Each week all participants will receive a text-message question regarding the time spent on exercises performed for their shoulder disorder. Participants in the intervention group will be instructed not to include the time spent on the add-on intervention in this report.

In the intervention group, face-to-face adherence reminders will be given by the investigator administering the intervention at the initial intervention instruction and at each subsequent intervention appointment (week 0, 2, 5, 10 and 16). The reminders will focus on (1) the importance of performing all prescribed exercises and on correct execution of exercises; (2) emphasising that the intervention is an add-on to usual care, and should not be a substitution for this; and (3) counteracting important barriers to ongoing engagement with home exercises, as identified by Littlewood et al. [42], namely the simplicity of the intervention (lack of potential effectiveness), lack of an early appreciable symptom response, when symptoms are reduced to a certain point, and a lack of self-efficacy.

Intervention adherence will further be enhanced by supplying a leaflet with the programme to stick on the fridge, and via the proactive follow-up intervention appointments, aspects known to improve patients adherence to a home-based exercise programme [42]. For an English version of the leaflets, see Additional files 4, 5 and 6.

#### Interventions – concomitant care

Concomitant use of corticosteroid injections will be based on the treating orthopaedic specialist’s evaluation, which is in accordance to the departments standard procedure and in line with the Danish national guidelines for treatment of SIS [10]. For pain relief, concomitant use of orally suspended NSAIDs will be permitted at patients’ own discretion. Corticosteroid injections and use of pain medication will be recorded.

### Outcomes

#### Primary outcome

The primary outcome will be the change in the patient-reported outcome (PRO) score SPADI [43]. SPADI is a widely used shoulder-specific PRO [44], considered one of the most responsive shoulder PROs [45–48], with a minimal clinical important difference (MCID) of 8 to 13 points [45, 48] and a high standardised response mean of 1.38. The SPADI is easy and fast to complete [48] and, in a recent systematic review [49], it was highlighted as one of three PROs for patients with rotator cuff disease for which the psychometric properties are supported by most strong or moderate evidence.

Baseline values for the primary outcome will be collected from patient records, and passed on by the treating physician to the primary investigator, as completion of the SPADI questionnaire is an integral part of the medical examination. Primary outcome data will be further collected at follow-up weeks 5, 10 and 16, with the main analysis conducted for changes from baseline to 16 weeks follow-up, reported as the difference in mean change between groups.

#### Secondary outcomes

**Fig. 1:**
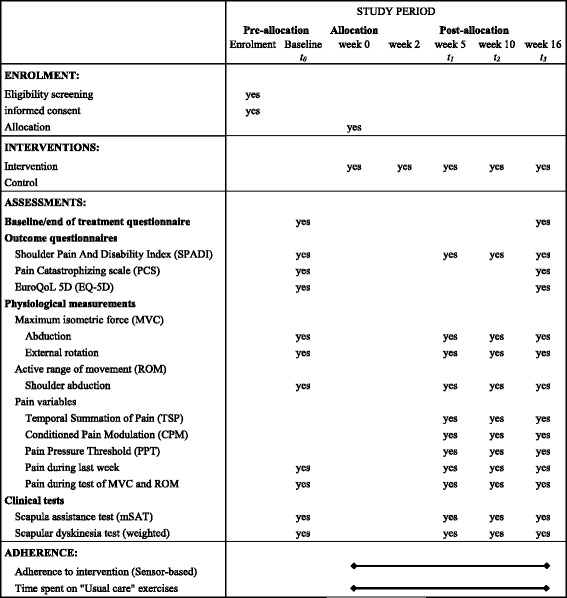
Summary of measures to be collected (SPIRIT figure)

Secondary outcome data will be collected at one or more of the time-points (1) baseline assessment, (2) 5 weeks follow-up, (3) 10 weeks follow-up and (4) 16 weeks follow-up. See participant timeline in the SPIRIT diagram (Fig. 1) for further details.

For the following secondary outcomes, the main analysis will be conducted for changes from baseline to 16 weeks follow-up, reported as the difference in mean change between groups.

- Maximum isometric voluntary contraction (MVC) in abduction and external rotation measured using a handheld dynamometer in neutral position (torque in Newton meter per kilo body weight, Nm/kg). Measures of strength relate directly to the intervention.
- Range of movement (ROM) in active shoulder abduction (0 to 180°), measured using a digital inclinometer, a widely used measure of function in patients with SIS.
- An average of least pain and average pain experienced during the last week (Numeric Rating Scale: 0–10), a valid and reliable measure of pain [50]. A measure of pain is important, as pain is one of the cardinal complaints associated with SIS [51].
- Pain catastrophizing (score from 0 to 52), being exaggerated negative thoughts related to experienced or anticipated pain, which might be a risk factor for chronicity [52], measured using the Danish version of the Pain Catastrophizing Scale.
- Health-related Quality of Life index (EQ-5D-index, range −0.167 to 1.00) measured with the EQ-5D-3 L [53].
- Health-related Quality of Life Visual Analogue Scale (EQ-5D visual analogue scale, score 0–100, 0 = lowest health-related quality of life) measured with the EQ-5D-3 L [53].

For the following secondary outcomes, the main analysis will be conducted for the outcome at 16 weeks follow-up, reported as the difference between groups at 16 weeks follow-up.

- Temporal Summation of Pain (TSP), a measure of central excitability (range 0 to 10 cm on electronic Visual analogue scale). A higher degree of TSP has previously been found in patients with unilateral SIS compared to healthy controls [54], and the decrease in TSP after surgical intervention is reported to be associated with the outcome of surgery [26].
- Conditioned Pain Modulation (CPM), a measure of the attenuated pain response (increase in tolerated pressure) to a painful pressure stimulus during application of another painful pressure stimulus [55]. CPM is a proxy measure of the function of the endogenous analgesia system, and patients with SIS have a lower CPM compared to healthy controls [54].
- Pressure Pain Threshold (PPT) is a measure of local mechanical hyperalgesia. It measures how much mechanical pressure is needed to elicit the first onset of pain. PPT is measured in kPa as the threshold for first detection of pain. Patients with SIS have lower PPTs in the shoulder region when compared to matched controls, possibly indicating peripheral sensitisation of the nervous system [26].
- Scapular Dysfunction (yes/no), measured using the modified Scapula Assistance Test, assessing if scapula muscle dysfunction influences the shoulder disorder. The result of the modified Scapula Assistance Test is a judgement of either positive or negative.
- Scapula Dyskinesia (yes/no), measured using the Scapula Dyskinesis Test, the result of which is a judgment of either positive or negative test.
- Global Impression of Change, measured on a 7-point Likert scale ranging from ‘Much better, a very important improvement’ to ‘Much worse, an important aggravation’.
- Patient Acceptable Symptom State, measured as the dichotomous answer (yes/no) to a standardised question regarding the acceptability of the current state of the shoulder symptoms.

Other outcomes of interest will be pain during testing of external rotation MVC, abduction MVC and active abduction ROM, as reported by the participant on a numeric pain rating scale (0 to 10, 0 = no pain), using standardised verbal anchors for the instruction [50].

### Sample size

The sample size estimation is based on the primary outcome SPADI. Studies on patients with SIS have shown standard deviations (SD) for SPADI change score after 11–12 weeks of between 17 [12] and 18.5 [56], while longer durations seem to entail larger SDs, with a SD of 22 reported for SPADI change from 0 to 22 weeks [12]. The latter is similar to our own unpublished data from SIS patients, showing a SD of 22.5 for SPADI change from 0 to 6 months. Based on this previous data, we expect a common SD of 19.5 for SPADI change values from week 0 to 16, the primary outcome. For the purpose of this study, the MCID for SPADI will be considered to be 10 points, based on previous studies [45, 48]. The negative effect that any dropouts will have on the statistical power will, to some degree, be reduced by the use of multiple imputation. However, the imputation of data will not fully redeem this, as multiple imputation can cause an underestimation of the effect and a larger variation in outcomes. Therefore, we aim at having a high power of 95% to verify an effect equal to or higher than the MCID of 10 points on SPADI, at a 5% significance level. To obtain this, a total of 200 patients will be required (100 in each group). This corresponds to a power of 89.7% in case of a 20% dropout and a power of 85.4% in case of a 40% dropout.

### Recruitment strategies

All participants will be recruited consecutively from the arthroscopic centre at the orthopaedic department, Hvidovre Hospital immediately after undergoing clinical examination performed by an orthopaedic specialist, who will also make the initial eligibility screening and provide written information about the trial for eligible patients. In order to achieve adequate enrolment, the orthopaedic specialists, who are at the first line of recruitment, will be informed about the progress of the trial at regular formal and informal meetings.

### Procedures and data collection methods

At baseline, after written consent is signed and before randomisation, all baseline assessments (Fig. 1) will be conducted by clinical physiotherapists who are trained in all assessment procedures and mutually aligned in order to improve the quality of data. All post-allocation assessments (week 5, 10 and 16) will be performed by the same group of testers in order to secure consensus regarding the instructions in questionnaires and conduction of tests. Participants will be instructed not to take any pain medication during the 8 hours prior to any assessment. Outcome assessors will also be trained in counselling for adherence to follow-up testing (to facilitate retention) and avoidance of disclosing allocation in a uniform manner. The study instruments are all described in detail in Additional file 7.

In general, once a participant is enrolled, every reasonable effort will be made to collect all outcomes for that participant, regardless of any deviations from the intervention protocol. Participants will receive text-message reminders for all scheduled follow-up appointments. The weekly text-messages with standardised questions regarding exercise time will also ensure that participants keep in mind their participation in the study. If participants do not attend their scheduled follow-up assessment, they will be contacted and offered to re-schedule to another date. If a participant should withdraw consent to follow-up assessments, the participant is offered the option to continue with the assessment of PROs.

Reasons for non-adherence to add-on intervention will be recorded by the investigator providing the intervention, either at the intervention visits or by phone if the participant does not come to the scheduled appointment. Reasons for non-retention will be recorded by the outcome assessor if this participant withdraws during a follow-up session, or by the primary investigator in a telephone interview.

#### Data management

All patient-reported questionnaires (SPADI, Pain Catastrophizing Scale, EQ-5D-5 L, Baseline and End of treatment) will be filled in by the patient in a paper format, and all data from the physiological measurements and clinical tests will be entered in a paper Case Report Form. Subsequently, all data will be entered in EpiData (version 3.1 or newer) by study personnel, using blinded double data entry to ensure data quality. The data entry form will support valid values and range checks where applicable. The original forms will be kept on file at a secure location on the study site for a period of 3 years after completion of the study. Data collected through SMS-track is entered directly by the participant as they send the answer in a text-message. The validity of data will be secured through answer validation, where answers that do not fit the specified requirements will be rejected. Reminder messages will be automatically generated in case of missing answers.

All data from the BandCizer units will be stored on the unit and transferred the BandCizer Backend Software (BandCizer^®^, Denmark) via Bluetooth connection to a dedicated repeater unit with internet access. All electronically entered data will be stored on a secure drive at the study site.

No data monitoring committee will be composed and no formal stopping guidelines and corresponding interim analyses are planned. No other interim analyses are planned.

### Allocation

Participants will be randomly assigned to either the control or intervention group (CG or IG) at a 1:1 allocation ratio, using a computer generated randomisation schedule of permuted blocks of random sizes ranging from 4 to 10.

Participants will be randomised using sequentially numbered, opaque, sealed envelopes. Investigators taking part in allocation and data collection will be blinded to block sizes and randomisation sequence at all times during the study period. Allocation concealment will be ensured, as the envelopes will not be opened before the participant has been irreversibly included in the study.

The creation of the randomisation schedule of permuted blocks of random sizes, and subsequent packaging of sequentially numbered, opaque, sealed envelopes will be performed by persons not else involved in the trial.

The final enrolment and subsequent allocation of participants will be conducted by investigators not taking part in any outcome assessment, who will be blinded to the randomisation sequence at all times during the intervention period. Outcome assessors will not take part in any of the processes related to allocation.

### Blinding

Outcome assessors performing the outcome assessment at baseline and follow-up weeks 5, 10 and 16 will be blinded to group allocation. Given the nature of the intervention, which requires the treating therapist to know the intervention, blinding of intervention providers is not deemed feasible, and therefore will not be performed. In order to obtain valid results from this trial, intervention receivers will be kept blind to treatment allocation. This will be attained through minimal information about the content of the add-on intervention and control condition until group allocation is final. Accordingly, participants will be informed (written and orally) that the study compares two different treatment regimens, and that both include the treatment elements normally offered, and adhere to the clinical guideline. Additionally, study participant will be strongly inculcated not to enclose or discuss treatment allocation with the outcome assessors, medical doctors or physiotherapists providing usual care.

All pre-defined analyses will be performed by a data analyst blinded to allocation.

#### Emergency unblinding

Investigators and caregivers will be encouraged to consult with the Medical Advisor (PH) in a case where unblinding seems relevant. As the intervention is only an add-on to usual care, and it will be modified with respect to the participants pain response, the probability of circumstances occurring where unblinding could be relevant seem very slim.

### Statistical methods – outcomes

For the primary end-point, namely SPADI score after 16 weeks, and for all continuous secondary outcomes listed in Table 2, a constrained linear mixed model (cLMM) will be applied in order to compare the change from baseline to 16 weeks in the IG to that in the CG. The model will contain the outcome at 16 weeks as the dependent variable, treatment group (IG or CG) as the main effect and both baseline score and any additional follow-up measurements as repeated measurements, to estimate the differences in mean change between groups and corresponding 95% confidence intervals (95% CI) (Table 3). The covariance structures will be selected based on the MAICE procedure [57].

**Table 2:** Variables, outcome measures and methods of analysis

Variable/outcome	Hypothesis	Outcome measure	Methods of analysis
Primary outcome	Intervention improved outcome		
SPADI change Δt_0_-t_3_		Score 0–100 (continuous)	cLMM
Secondary outcomes	Intervention improved outcome		
Abd. MVC change Δt_0_-t_3_		Nm/kg (continuous)	cLMM
Ext. rot. MVC change Δt_0_-t_3_		Nm/kg (continuous)	cLMM
Abd. total ROM change Δt_0_-t_3_		Degrees (continuous)	cLMM
Pain last week change Δt_0_-t_3_		NRS 0–10 (continuous)	cLMM
EQ-5D change Δt_0_-t_3_		Index value (continuous)	cLMM
Global impression of change, at t_3_		% much improved/fully recovered (binary)	χ^2^ test
Pain sensitisation Δt_1_-t_3_			
Temporal Summation of Pain		VAS increase (continuous)	cLMM
Conditioned Pain Modulation		kPa increase (continuous)	cLMM
Pain Pressure Threshold		kPa (continuous)	cLMM
Scapular involvement			
SAT		% positive (binary)	χ^2^ test
SDT		% positive (binary)	χ^2^ test
Subgroup analyses			
Scapula involvement (SAT and SDT yes/no)	Dysfunction modifies the effect of intervention	SPADI score, abduction MVC and external rotation MVC (continuous)	cLMM with interaction term
Central sensitisation (TS and CPM high vs. low)	CS modifies the effect of intervention	SPADI score, abduction MVC and external rotation MVC (continuous)	cLMM with interaction term
Sensitivity analyses	Intervention improved outcome	Primary outcome	
Per protocol analysis			cLMM
Adjusting for baseline			cLMM

*cLMM* constrained linear mixed model, *EQ-5D* EuroQol 5D-3 L, *MVC* maximum voluntary contraction, *Nm/kg* Newton meter per kilo body weight, *ROM* range of movement, *SAT* scapula dysfunction, *SDT* scapula dyskinesia, *SPADI* Shoulder Pain and Disability Index, VAS visual analogue scale

**Table 3:** Outcomes (Mean, SD)

	Week 0		Week 5		Week 10		Week 16	
	IG	CG	IG	CG	IG	CG	IG	CG
SPADI score								
Abduction MVC								
External rotation MVC								
Abduction ROM								
Pain last week								
QoL (EQ-5D)								

*CG* control group, *IG* intervention group, *MVC* maximum voluntary contraction, *QoL* quality of life, *ROM* range of movement, *SPADI* Shoulder Pain and Disability Index

**Fig. 2:**
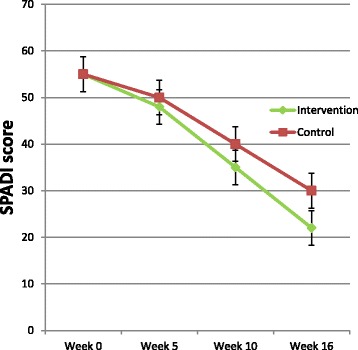
Visualisation of changes in SPADI score in the intervention and control group, respectively (example, not based on data)

Mean scores and corresponding 95% CIs will be reported for all outcome time-points (t_0_, t_1_, t_2_ and t_3_) (Table 3). Within group changes between all outcomes assessment time-points (t_0_-t_1_, t_1_-t_2_ and t_2_-t_3_), and between baseline and last assessment (t_0_ and t_3_), and corresponding 95% CI, will be reported (Table 4). Finally, differences in within group changes between all outcomes assessment time-points (t_0_-t_1_, t_1_-t_2_ and t_2_-t_3_) and the corresponding 95% CIs will be reported (Table 5). Results from the repeated measures analysis with SPADI score as outcome will be visualised as illustrated in Fig. 2.

**Table 4:** Within group change scores (95% CI)

	Week 0–5		Week 5–10		Week 10–16		Week 0–16	
	IG	CG	IG	CG	IG	CG	IG	CG
SPADI score								
Abduction MVC								
External rotation MVC								
Abduction ROM								
Pain last week								
QoL (EQ-5D)								

*CG* control group, *IG* intervention group, *MVC* maximum voluntary contraction, *QoL* quality of life, *ROM* range of movement, *SPADI* Shoulder Pain and Disability Index

**Table 5:** Between group difference in change scores (95% CI)

	Week 0 to 5 (95% CI)	Week 5 to 10 (95% CI)	Week 10 to 16 (95% CI)	Week 0 to 16 (95% CI)
SPADI score				
Abduction MVC				
External rotation MVC				
Abduction ROM				
Pain last week				
QoL (EQ-5D)				

*CG* control group, *IG* intervention group, *MVC* maximum voluntary contraction, *QoL* quality of life, *ROM* range of movement, *SPADI* Shoulder Pain and Disability Index

For comparison of binary outcomes, proportions will be compared using a χ^2^ test, and odds ratio estimates and corresponding 95% CIs will be reported.

Results from analyses comparing IG and CG for all primary and secondary outcomes will be reported in the second paper outlined in the ‘Dissemination policy’ section, except for the results on secondary outcomes regarding pain sensitisation and scapula dyskinesia, which will be reported in the third and fourth paper outlined in the ‘Dissemination policy’ section, respectively.

*P* values will be reported to the fourth decimal. For all tests, a two-sided significance level of ≤ 0.05 will be applied. All analyses will be conducted using up-to-date versions of SPSS and SAS.

#### Statistical methods – additional analyses

##### Subgroup analyses

For the outcomes SPADI score, abduction MVC and external rotation MVC, subgroup analyses investigating the modifying effect of scapula dysfunction, scapula dyskinesia, temporal summation of pain, conditioned pain modulation, pain pressure threshold (site 1) and pain catastrophizing, respectively, will be conducted. These will be performed using a cLMM, similar to the corresponding main analyses, but including a dichotomised value for the first measurement of the relevant variable as the interaction term and reporting the effect estimates for each subgroup.

The following sensitivity analyses will be performed for the primary outcome and reported together with the primary analyses in the primary trial report:

- A per protocol analysis, similar to the primary analysis, will be performed. From the IG, only patients who attended intervention appointments on weeks 0, 5 and 10 will be included in this analysis, while all patients in the CG will be included.
- Adjusted cLMM to adjust the effect of treatment allocation on SPADI score for any baseline differences between groups. Baseline variables will be included as covariates if (1) the difference between groups is more than 0.3 of the common SD for continuous outcomes, or (2) proportions are significantly different (*P* < 0.05) between groups, for binary outcomes.
- Sensitivity analyses to investigate the importance of the clinometric properties of the SPADI score may be relevant. This is based on a very recent study [58], which evaluates the SPADI score using the Rasch model. The study was published after initiation of this trial.

Three exploratory dose-response analyses will be performed. Only participants in the IG will be included in these analyses. First, the interacting effect of adherence to the intervention, TSP and CPM, on change in external rotation MVC, abduction MVC and SPADI score (outcomes), will be investigated. Secondly, whether the effect of TSP and CPM is mediated by adherence to the intervention will also be assessed. These analyses will be conducted as available-case analyses.

For all included variables, data from each time-period (t_0_-t_1_, t_1_-t_2_ and t_2_-t_3_) will be included as repeated measurements in these analyses. The outcome variable is the change between first and last measurement in a time-period. ‘Adherence’ to the intervention is the total TUT in a time-period. ‘Central Pain Sensitization’ score (TSP or CPM) is the score from the last time-point in each time-period.

Additionally, results will also be presented for similar analyses adjusted for Usual Care, calculated for each time-period as the average of time spent on usual care, as reported by text-message, in all weeks included in the relevant time-period.

#### Statistical methods  – analysis population and missing data

All main analyses will be conducted as intention-to-treat analyses, including all randomised participants, regardless of protocol adherence. Participants will be analysed as randomised. To create a full analysis dataset for the intention-to-treat analyses, missing outcome data will be imputed using multiple imputations based on the variables of all previous scores in the relevant outcome, age, sex and allocation.

### Harms

Adverse events will be defined in this context as any unintended, unfavourable findings, symptom or illnesses that occur during the assessment or the add-on intervention, whether it can be attributed to the assessment or not. Adverse events will be recorded in part by the patient as a spontaneous recording during assessment and by open questioning.

Acute exacerbations of shoulder symptoms will be recorded by the primary investigator (MC) and, as a safety precaution, in case a medical evaluation is required, the participant will be referred to the Medical Advisor (PH). Patients in the intervention group experiencing exacerbations lasting more than 1 week will be referred to the Medical Advisor (PH) and evaluated if further medical care is needed.

Serious unexpected side effects or serious adverse events will be reported to the Capital Regional Ethics Committee in Denmark within 7 days after sponsor or the primary investigator has become aware of the incident. Serious adverse events will be categorised according to the definitions established by the United States Food and Drug Administration [59] and will be assessed by the primary investigator for possible relations with the assessment and/or intervention to consider whether there is a reasonable possibility that the adverse event can be caused by either.

No audits are planned.

### Dissemination policy

All results from the study, be they positive, negative or inconclusive, will be published in international scientific journals. The project leader will enforce publication. Furthermore, the results will be presented at national and international conferences. Working titles for scientific publications are listed below. In the primary trial report, all collected outcomes will be defined and referenced to the dissemination plan below if data or analyses are not reported in the primary trial report.

1. The Strengthening Exercises in Shoulder Impingement trial (The SExSI-Trial): Protocol for a pragmatic, assessor blinded, parallel-group, randomised, controlled, superiority trial investigating the effectiveness of adding a simple shoulder strengthening exercise programme to usual care, in patients with long-lasting subacromial impingement syndrome.
2. The effectiveness of adding to usual care a simple programme of Strengthening Exercises In Subacromial Impingement patients (The SExSI-Trial): A randomised controlled trial (Primary trial report focusing on the primary outcome and analysis, as well as supportive secondary analyses for the primary outcome).
3. The dose-response relationship between adherence to the intervention and changes in shoulder strength and patient-reported shoulder function: Pre-defined secondary analyses from the SExSI-Trial.
4. The effectiveness of the SExSI-Trial add-on intervention on pain sensitisation, the modifying effect pain sensitisation on treatment outcomes and mediating effects of pain sensitisation on the dose-response relationship between intervention adherence and changes in outcomes: Pre-defined secondary analyses from the SExSI-Trial.
5. The modifying effect of scapula dysfunction at baseline on shoulder strength and function outcomes and the effectiveness of the add-on intervention on scapula dysfunction: Pre-defined secondary analyses from the SExSI-Trial.

Additionally, the results of this study will be communicated directly to the participants, who will be encouraged to comment on disseminated trial results in order to improve their further public dissemination. Furthermore, trial results will be disseminated to the public in general through the daily press.

#### Dissemination policy – authorship

Decisions on authorship eligibility will adhere to the Harvard author guideline statement as endorsed by the Faculty Council of Harvard Medical School (https://hms.harvard.edu/sites/default/files/assets/Sites/Ombuds/files/AUTHORSHIP%20GUIDELINES.pdf).

Topics suggested for presentation and/or publication, including suggestion and justification for authors to be reviewed for the Writing Committee, will be presented to the members of the Steering Committee. The Steering Committee will form the Writing Committee and decide on author sequence. Disputes regarding authorship will be settled by the Primary investigator after consulting with the Supervising investigator.

## Discussion

This trial (the SExSI-Trial) will investigate the effectiveness of adding a simple shoulder strengthening exercise programme to usual care in patients with long-lasting SIS. If the intervention is found effective in improving patient-reported shoulder function, the intervention will be easily implemented as an addition to usual care.

The current trial is planned and designed to maximise the usefulness of the trial results by focusing on features that have recently been suggested as important when considering the usefulness of clinical research [60]. We further believe that the research question raised in this trial satisfies the FINER-criteria [61], as it is considered both Feasible, Interesting, Novel, Ethical and Relevant (Table 6).

**Table 6:** Features to consider in appraising whether clinical research is useful (retrieved from Ioannides [60])

Feature	Question to ask
Problem base	Is there a health problem that is big/important enough to fix?
Context placement	Has prior evidence been systematically assessed to inform (the need for) new studies?
Information gain	Is the proposed study large and long enough to be sufficiently informative?
Pragmatism	Does the research reflect real life? If it deviates, does this matter?
Patient centeredness	Does the research reflect top patient priorities?
Value for money	Is the research worth the money?
Feasibility	Can this research be done?
Transparency	Are methods, data and analyses verifiable and unbiased?

Firstly, the ‘problem base’ and ‘context placement’ have been described in the Background section. Accordingly, the treatment of shoulder impingement syndrome is a problem that is worthwhile to address, based on the extent of the problem and the substantial societal costs related to the disorder. Furthermore, the systematic literature review, as is presented in the Background section, clearly indicates that knowledge is lacking with regards to the effect of strengthening exercises in the rehabilitation of patients with long-lasting SIS. The SExSI-Trial will provide relevant insight into this area, contributing to fill this gap.

Secondly, a focus on information gain, pragmatism and patient centeredness is secured through methodological considerations. The trial has been accordingly designed to optimise the information gained from the study by implementing multiple follow-up time-points and a close monitoring of intervention adherence. This makes it possible to conduct relevant dose-response analyses, which in turn will guide future research. The pragmatism of the trial is improved by the use of broad eligibility criteria, a consecutive sampling strategy and usual care as the comparator, aspects that will improve the generalisability of the trial results. Moreover, the choice of SPADI as the primary outcome is in line with the fact that pain and loss of function are the main complaints associated with SIS [51]. This improves the patient centeredness of the trial, as the goal of the intervention is meaningful to the patients.

Finally, the pre-registration at clinicaltrials.gov and publication of this trial protocol, including intervention descriptions based on the TIDieR framework [33], greatly improves the transparency of the trial conduct and results. Collectively, all of these efforts are likely to improve the usefulness of the trial results, and hence the relevance of the trial itself.

### Strengths and limitations

Aside from the above, this trial has some methodological strengths and limitations which must be taken into account when interpreting the study findings.

#### Strengths

The blinding of participants, data analysts, outcome assessor and usual care providers greatly decreases the risk of bias in the current trial, supporting the internal validity of study findings. The external validity is further improved by the use of consecutive sampling, thereby improving the generalisability of the results.

#### Limitations

Given the nature of the intervention, the intervention providers could not be blinded, which increases the risk of bias in the trial. Furthermore, the use of a single centre study design, and the fact that the intervention is provided in a setting outside the normal usual care, decreases the external validity of the study, as study findings might not be generalisable to other settings. It is not within the scope of this study to conduct cost-effectiveness analyses or to conduct qualitative work to evaluate patient acceptability and experience of the home-based exercise programme. While the objective monitoring of adherence will show to what degree patients have accepted and managed to perform the intervention as prescribed, qualitative work could be conducted in order to investigate patient experiences with the intervention and reasons for adherence and non-adherence in order to improve implementation of the intervention. Cost-effectiveness analyses could also be performed in order to inform policy-makers and support implementation. The current study will, nevertheless, provide level-one evidence regarding the effectiveness of the add-on intervention.

## Trial status

### Protocol version

Issue date: 10 May 2017, Protocol amendment 01, Author MC.

#### Revision chronology

Version 1, 25.02.2016 Original.

Version 2, 10.05.2017 Amendment 1. Primary reason for amendment: EQ-5D-3 L was added as outcome at 1-year follow-up.

#### Recruitment

Recruitment was initiated May 1, 2016, and is expected to be finalised April 1, 2018.

## Additional files

Additional file 1: Populated SPIRIT checklist (PDF 172 kb)

Additional file 2: TIDieR control, usual care. (PDF 113 kb)

Additional file 3: TIDieR intervention, strengthen your shoulder. (PDF 207 kb)

Additional file 4: Strengthen your shoulder, intervention leaflet 1. (PDF 411 kb)

Additional file 5: Strengthen your shoulder, intervention leaflet 2. (PDF 557 kb)

Additional file 6: Strengthen your shoulder, intervention leaflet 3. (PDF 588 kb)

Additional file 7: Data collection methods – study instruments. (PDF 235 kb)

## Abbreviations

CG: control group
cLMM: constrained linear mixed model
CPM: conditioned pain modulation
IG: intervention group
MCID: minimal clinical important difference
MVC: maximum isometric voluntary contraction
PPT: pressure pain threshold
PRO: patient-reported outcome
ROM: range of movement
SExSI: Strengthening Exercises in Shoulder Impingement
SIS: subacromial impingement syndrome
SPADI: Shoulder Pain And Disability Index
TSP: temporal summation of pain
TUT: time under tension
