# Supplementary material for: The Strengthening Exercises in Shoulder Impingement trial (The SExSI-trial) investigating the effectiveness of a simple add-on shoulder strengthening exercise programme in patients with long-lasting subacromial impingement syndrome: Study protocol for a pragmatic, assessor blinded, parallel-group, randomised, controlled trial
Source: Trials. 2018 Mar 2;19:154. doi: 10.1186/s13063-018-2509-7 (PMC5833202; doi:10.1186/s13063-018-2509-7)
Supplement: Supplementary file 2 — TIDieR control, usual care. (PDF 113 kb) [file 13063_2018_2509_MOESM2_ESM.pdf]

Usual care in Denmark during the SExSI-Trial:

- A description of usual care including all relevant TIDieR Items (Item 1)

Rationale (Item 2)
The rationale for using usual care as the comparator is explained in the main study protocol.

Materials (Item 3)
A “Rehabilitation Plan” is send from the treating orthopedic specialist to the relevant Rehabilitation Center, which is responsible for contacting the patient. During rehabilitation, the treating therapist may decide to provide the patient with written descriptions of exercises.

Procedures (Item 4)
Patients are offered a referral to general rehabilitation in the municipal under the Danish health Act § 140, sometimes with the option to choose a private physiotherapy clinic, partly at their own expense instead. The referral is standard procedure when a patient with SIS is considered to have a medically justified need for general rehabilitation, as identified during the examination at the orthopaedic outpatient department (secondary care unit). This procedure is based on the Danish National clinical guideline on diagnostics and treatment of patients with selected shoulder disorders (10), as exercise therapy is recommended as first line of treatment for SIS.
In the context of this study, usual care includes all treatment received by a patient during the time between baseline and follow-up, except that included in "Strengthen your Shoulder". Therefore, usual care might include a range of treatment modalities including advice, stretching, exercises, manual therapy, massage, acupuncture and electrotherapy at the discretion of the treating physiotherapist and doctor.

Providers (Item 5)
Usual care is provided by the healthcare professionals employed in the relevant institutions and/or physiotherapy clinics, with no interference from the investigators.

How, Where, When and How much (Items 6-8)
Decisions on how, when and where usual care is provided will be made with no interference from the investigators. Most often, general rehabilitation will be initiated in a municipality rehabilitation centre in the municipal of residence. First contact from the centre to the patient often takes place within two weeks after referral from the hospital. All following sessions are planned in collaboration between the patient and the treating physiotherapist. The amount of rehabilitation sessions offered to the patient depends on how the legislative framework for general rehabilitation of shoulder disorders is implemented in the municipality of residence, with the number of possible sessions most often varying from two per week to once per month within the first 2-4 months after referral .

As an example, in some municipalities, patients are granted four hours of individual rehabilitation in total. These can then be used for rehabilitation in small groups instead, resulting in approx. 16 group sessions over a period of 7-8 weeks. Alternatively, the four hours can be used for individual sessions. It should be noted, however, that the implementation of the legislative framework likely differs considerably between municipalities.

Tailoring (Item 9 and 10)
For all patients, usual care is tailored and modified at the discretion of the treating physiotherapist or doctor.

Adherence and fidelity (Item 11).
The time spend on exercises related to usual care is monitored by self-report, using an SMS-track system. Each week all participants will receive a text-message question regarding the time spent on exercises performed for their shoulder disorder. Participants in the intervention group will be instructed not to include the time spent on the add-on intervention in this report. Furthermore, at each follow-up visit, the number of physiotherapy sessions, doctor visits, steroid injections and use of pain medication related to the shoulder disorder will be recorded.

This is an Additional File for the article titled:
The Strengthening Exercises in Shoulder Impingement trial (The SExSI-trial) investigating the effectiveness of a simple add-on shoulder strengthening exercise programme in patients with long lasting subacromial impingement syndrome: Study protocol for a pragmatic, assessor blinded, parallel-group, randomised, controlled trial
