# Supplementary material for: The Strengthening Exercises in Shoulder Impingement trial (The SExSI-trial) investigating the effectiveness of a simple add-on shoulder strengthening exercise programme in patients with long-lasting subacromial impingement syndrome: Study protocol for a pragmatic, assessor blinded, parallel-group, randomised, controlled trial
Source: Trials. 2018 Mar 2;19:154. doi: 10.1186/s13063-018-2509-7 (PMC5833202; doi:10.1186/s13063-018-2509-7)
Supplement: Supplementary file 3 — TIDieR intervention, strengthen your shoulder. (PDF 207 kb) [file 13063_2018_2509_MOESM3_ESM.pdf]

Strengthen your Shoulder:

- An intervention description for the SExSI-Trial including all TIDieR Items (Item 1)

Rationale (Item 2)

The rationale for the intervention and the theory supporting it is described in the main study protocol.

Materials (Item 3)

Three separate leaflets were developed, one for each phase of the intervention program. The leaflets contains:

- A short description of the aim, content and rationale of the intervention as a whole and the specific phase of the program (Phase1, week 1 to 5; Phase 2, week 6-10; Phase 3, week 11-16).
- Pictures and written bullet points for descriptions of the relevant exercises and strength training descriptors.
- Information on rules for progression and regression of exercises and corresponding algorithm.
- Statement regarding known advantages, disadvantages and risks related to the intervention.
- Contact details on the primary investigator.
- Place of development and date of most recent revision

Latex free elastic exercise band sheets from Thera-Band®, cut in approximately 220 cm length and fastened in a loop using a plastic clip.
A chair and a table is used for Exercise 1.

Links to instructional videos.
Available upon publication
of the primary trial report.

Procedures (Item 4)

Patients in the intervention group will, in addition to usual care, receive instructions in a home-based, progressive, high volume resistance training programme.

First, instructions to the aim and rationale of the intervention are provided orally, supported by the information leaflet. The following points are covered in this information:

- Strength training is needed to increase capacity (the shoulder is able to endure more without causing any trouble)
- Exercises with long isometric holds reduce the peak load (safe) while ensuring a high volume (needed)
- Pain is OK, as long as it is bearable. This is not a sign of danger.
- The pain system can be affected because of long lasting pain.
  - Normal function of the pain system is to warn about potential damage.
  - With long lasting pain, the pain system is potentially and likely not functioning normally and hence is not a liable indicator of potential damage.
- As an indicator of whether training load is appropriate, instead of pain during the exercises, the flare up of symptoms following exercises is used to guide the need for regression of exercises. This means that a flare up of symptoms lasting for more than 24 hours indicates that training load has been too high, and exercise load should be decreased the next time exercises are performed.
- As a side effect, using this approach could possibly help rehabilitate the pain system.
- Additionally, some have reported that using exercises with long (isometric) holds may reduce pain, reportedly to the same degree as a regular painkiller.

The program

The programme will consist of three phases; Phase 1 (week 0-5), Phase 2 (week 6-10) and Phase 3 (week 11-16), and include three exercises, starting with one exercise in Phase 1 and adding one additional exercise at Phase 2 and 3, respectively. The exercises are; i) external rotation with the elbow supported in app. 45 degrees shoulder scaption, ii) abduction with a slight degree of scaption to app. 45 degrees, and iii) external rotation with the elbow unsupported in app. 45 degrees scaption, all exercises performed with elastic band as external resistance. Exercises are performed with a 1-2 sec. concentric contraction, 5 sec. isometric hold in the end-position and 1-2 sec. eccentric contraction and repetitions to failure.

The relative resistance in all exercises are aimed at 15-20 RM in Phase 1, 10-15 RM in Phase 2 and 8-10 RM in Phase 3. In order to determine the starting resistance for the initiation of the intervention (15 RM), a testing session will be conducted, and the endpoints on the elastic band to be used are marked so that it is easy for the patient to replicate the exercise with the same resistance at home. The patients are instructed to adjust the resistance by shortening the distance between the endpoints approximately 5 cm when they are able to perform more repetitions than the highest value for the relevant step (20, 15 and 10 repetitions, respectively). The relative resistance will be adjusted at each follow-up visit. Patients will be instructed to keep a good posture with the shoulders back and straighten their back when performing exercises. Patients are also instructed that, in case of a missed exercise session, all future sessions is simply postponed by one day (e.g. When exercise sessions are planned every other day and one session is missed, that session is performed the next day instead, followed by one day with no exercise session etc.).

Upon publication of the primary trial report, instructional videos of the three exercises will be made available via the QR code found on the title-page of this Additional File.

Exercise 1: Supported external rotation

<u>Starting position:</u> The patient is seated on a chair with the affected side towards a table with the back straight, shoulders back and the elbow resting on the table with approximately 30-45 degrees of shoulder scaption. The affected arm is held in 90 degrees of shoulder internal rotation and 90 degrees of elbow flexion. The elastic band loop is held with the hand of the affected shoulder in one end, while the other end is fixated under the opposite foot.

<u>Movement:</u> Keeping all other body parts as in the starting position, the shoulder is externally rotated approximately 80 degrees in a 2 sec concentric contraction, the end-position is held for 5 seconds and finally the starting position is resumed via a 2 sec eccentric contraction. The end-position is defined as the position in which the largest resistance to external rotation is felt. With a resting period of 2 sec between repetitions, the exercise is repeated to failure.

Exercise 2: Standing elevation

<u>Starting position:</u> Patient is standing with the feet shoulder wide with the back straight and shoulders back with the affected shoulder in slight flexion and neutral rotation. The elastic band loop is held with the hand of the affected shoulder in one end, while the other end is fixated under the opposite foot.

<u>Movement:</u> Keeping all other body parts as in the starting position, the shoulder is abducted to approximately 45 degrees in a 2 sec concentric contraction, keeping a slight flexion, thereby allowing the elastic band move freely in front of the body. The end-position is held for 5 seconds and finally the starting position is resumed via a 2 sec eccentric contraction. With a resting period of 2 sec between repetitions, the exercise is repeated to failure.

Exercise 3: Unsupported external rotation

Starting position: Patient is standing with the feet shoulder wide with the back straight and shoulders back with the affected arm in approximately 45 degrees of shoulder scaption, 90 degrees of shoulder internal rotation and 90 degrees of elbow flexion. The elastic band loop is held with the hand of the affected shoulder in one end, while the other end is fixated under the opposite foot.

Movement: Keeping all other body parts as in the starting position, the shoulder is externally rotated approximately 45 degrees in a 2 sec concentric contraction, the end-position is held for 5 seconds and finally the starting position is resumed via a 2 sec eccentric contraction. The end-position is defined as the position in which the largest resistance to external rotation is felt. With a resting period of 2 sec between repetitions, the exercise is repeated to failure.

Providers (Item 5)

The intervention is primarily provided by the primary investigator, a physiotherapist with 5 years of clinical experience, trained in providing the intervention throughout the development phase and in pilot testing of the intervention. Alternates designated to take over in case the primary investigator is unable to complete one or more intervention sessions will be physiotherapists trained and approved by the primary investigator. Training will focus on uniform correction of exercise form, progression and regression of exercises and standard face-to-face adherence reminders.

How (Item 6)

The intervention (being instructions in the exercise program) is delivered face-to-face individually to each patient.

Where (Item 7)

The face-to-face instructions are provided at the study site, in a designated intervention room. Exercises are performed at home (or other location away from the study site).

When and how much (Item 8):

Five intervention sessions are planned. The first session is to be held as shortly after inclusion as possible, duration 30 minutes. Two weeks after the first session, an early adjustment session lasting 15 minutes is conducted. Five and ten weeks after inclusion, respectively, a session lasting 30 minutes focusing on progression of exercises and adjustment of exercise form is completed. Sixteen weeks after inclusion, an end of treatment session lasting 30 minutes is conducted.

Tailoring (Item 9)

For all intervention Phases, a standard protocol including adaptation algorithm based on pain response and repetition maximum increase is implemented, for individual progression/regression of resistance and exercises. Progression of resistance will be based on the number of repetitions that an individual is able to perform a given exercise, the repetition maximum (RM) principle. A standard protocol for regression of resistance and/or exercises will be based on pain response, instructing patients to stop exercises in case of flare-up of symptoms and to reduce resistance for the next session if the flare-up has lasted more than 24 hours. In case of flare up of symptoms lasting 24 hours or more and/or in case patients are unable to perform the minimum RM of the range specified for the given intervention step (i.e. less than 15 for Phase 1), the resistance is to be reduced by moving the mark on the elastic band to lengthen the part being stretched doing exercises by approximately 5 cm. In case the patient is able to perform more repetitions of the exercise then than specified for the given intervention Phase (i.e. more than 20 reps for Phase 1), the resistance is to be increased by moving the mark on the elastic band to shorten the part being stretched doing exercises by approximately 5 cm. When a patient is able to perform a number of reps included in the specified range for the given intervention Phase (i.e. 15 to 20 reps for Phase 1), the same resistance is to be used for the next exercise session.

Further individual adaptations of the exercises or the progression of exercises are made when:

1. A patient is unable to complete the full range of motion for a given exercise. Solution: In exercise 1 and 3, altered position of the glenohumeral joint (e.g. less static scaption) and/or less external rotation ROM during contraction. In Exercise 2, slight to moderate external rotation of the glenohumeral joint during the entire exercise and/or less scaption ROM performed during the concentric contraction.
2. A patient, for any reason, have not performed the intervention in a given Phase, and hence is not deemed fit to progress to the next level. Solution: The patient is not progressed to the next Phase of the intervention.

Modifications (Item 10)

In case of modifications to the intervention during the study period, these will be reported in the primary trial report.

Adherence and fidelity (Item 11)

Lack of adherence to an exercise intervention is a major problem when aiming at investigating the effect of an intervention. In the previously described studies that include unsupervised resistance training in rehabilitation of SIS patients, monitoring adherence is either done using a log-book filled in by the patients [1,2] or not described at all [3]. Using only self-reported measures of adherence may limit the possibility of detecting which exercise dose is actually received by the patient. Accordingly, in a recent systematic review, the authors did not identify a single measure of self-reported adherence to unsupervised home-based rehabilitation exercises, which was sufficiently investigated [4]. Furthermore, findings from one previous study revealed that the dose of exercises reported in exercise diaries were 2.3 times higher than that collected through a system, which monitored the exact time-under-tension (TUT), being the total time a muscle resists weight during each set [6]. This clearly underlines the relevance of collecting objective data on exercise adherence. In addition, it seems unlikely that patients are always able to "take" precisely the exercise dose prescribed. This issue is unfolded in a recent study, revealing that, just two weeks after initial instructions, only seven out of 29 young healthy individuals were able to use the correct TUT, when performing shoulder exercises with an elastic band [7].

In the SExSI-Trial, adherence to the add-on progressive high volume resistance training intervention is monitored using the BandCizer©. The BandCizer© is a small device which is mounted on the elastic band during exercises, and measures the time-under-tension (TUT), number of repetitions and total work load for all exercises performed. Exactly TUT is a promising objective measure of exercise adherence, for which a the BandCizer©, is specifically developed and validated [8–10]. By directly monitoring the actual TUT for the prescribed exercises, a more precise and objective distinguishing between adherent and less adherent patients will be possible.

Standard schemes for correction and monitoring of exercise form are used for each exercise at each follow-up visit. The following points are used:

Exercise 1

- Lower arm app. horizontal at the beginning of the exercise
- Elbow flexed to 90 degrees throughout the exercise
- Posture: Straight back and shoulders retracted (90 degrees flexion in hip is not required)
- Shoulder in 30-45 degrees of scaption
- Only movement in the shoulder
- Lower arm not in full vertical position when in the end position
- Able to adjust the elastic band

Exercise 2

- Posture: straight back and shoulders retracted
- Shoulder abducts to app. 45 dergress of scaption/abduction
- Elbow straight during the entire exercise

Exercise 3

- Lower arm app. horizontal at the beginning of the exercise
- Elbow flexed to 90 degrees throughout the exercise

- Posture: Straight back and shoulders retracted
- Shoulder in 30-45 degrees of scaption
- Only rotation movement in the shoulder
- Lower arm not in full vertical position when in the end position

In the intervention group, face-to-face adherence reminders will be given by the investigator administering the intervention at the initial intervention instruction and at each subsequent intervention appointment (week 0, 2, 5, 10 and 16). The reminders will focus on:

- The importance of performing all prescribed exercises and on correct execution of exercises.
- Emphasising that the intervention is an add-on to usual care, and should not be a substitution for this.
- Counteract important barriers to ongoing engagement with home-exercises identified by Littlewood et al. [11] , being simplicity of the intervention (lack of potential effectiveness), lack of an early appreciable symptom response, when symptoms are reduced to a certain point and a lack of self-efficacy.

Intervention adherence will further be enhanced by supplying a leaflet with the program to stick on the fridge, and via the proactive follow-up intervention appointments, aspects that improve patients adherence to a home-based exercise program [11]. For an English version of the leaflets, see Appendix 5, 6 and 7.

References

1. Bennell K, Wee E, Coburn S, Green S, Harris A, Staples M, et al. Efficacy of standardised manual therapy and home exercise programme for chronic rotator cuff disease: randomised placebo controlled trial. BMJ. 2010;340:c2756.

2. Holmgren T, Björnsson Hallgren H, Öberg B, Adolfsson L, Johansson K. Effect of specific exercise strategy on need for surgery in patients with subacromial impingement syndrome: randomised controlled study. BMJ. 2012;344:e787.

3. Maenhout AG, Mahieu NN, De Muynck M, De Wilde LF, Cools AM. Does adding heavy load eccentric training to rehabilitation of patients with unilateral subacromial impingement result in better outcome? A randomized, clinical trial. Knee Surg Sports Traumatol Arthrosc Off J ESSKA. 2013;21:1158–67.

4. Bollen JC, Dean SG, Siegert RJ, Howe TE, Goodwin VA. A systematic review of measures of self-reported adherence to unsupervised home-based rehabilitation exercise programmes, and their psychometric properties. BMJ Open. 2014;4:e005044.

5. Rathleff MS, Bandholm T, McGirr KA, Harring SI, Sørensen AS, Thorborg K. New exercise-integrated technology can monitor the dosage and quality of exercise performed against an elastic resistance band by adolescents with patellofemoral pain: an observational study. J Physiother. 2016;62:159–63.

6. Faber M, Andersen MH, Sevel C, Thorborg K, Bandholm T, Rathleff M. The majority are not performing home-exercises correctly two weeks after their initial instruction-an assessor-blinded study. PeerJ. 2015;3:e1102.

7. Rathleff MS, Bandholm T, Ahrendt P, Olesen JL, Thorborg K. Novel stretch-sensor technology allows quantification of adherence and quality of home-exercises: a validation study. Br J Sports Med. 2014;48:724–8.

8. Rathleff MS, Thorborg K, Rode LA, McGirr K, Sørensen AS, Bøgild A, et al. Adherence to commonly prescribed, home-based strength training exercises for the lower extremity can be objectively monitored using the Bandcizer. J Strength Cond Res Natl Strength Cond Assoc. 2014;

9. Skovdal Rathleff M, Thorborg K, Bandholm T. Concentric and eccentric time-under-tension during strengthening exercises: validity and reliability of stretch-sensor recordings from an elastic exercise-band. PloS One. 2013;8:e68172.

10. Littlewood C, Malliaras P, Mawson S, May S, Walters S. Patients with rotator cuff tendinopathy can successfully self-manage, but with certain caveats: a qualitative study. Physiotherapy. 2014;100:80–5.

This is an Additional File for the article titled:

The Strengthening Exercises in Shoulder Impingement trial (The SExSI-trial) investigating the effectiveness of a simple add-on shoulder strengthening exercise programme in patients with long lasting subacromial impingement syndrome: Study protocol for a pragmatic, assessor blinded, parallel-group, randomised, controlled trial
