# Supplementary material for: The Strengthening Exercises in Shoulder Impingement trial (The SExSI-trial) investigating the effectiveness of a simple add-on shoulder strengthening exercise programme in patients with long-lasting subacromial impingement syndrome: Study protocol for a pragmatic, assessor blinded, parallel-group, randomised, controlled trial
Source: Trials. 2018 Mar 2;19:154. doi: 10.1186/s13063-018-2509-7 (PMC5833202; doi:10.1186/s13063-018-2509-7)

Additional file 4, Strengthen your shoulder, leaflet, Phase 1

THE AIM of "Strengthen your shoulder":
The program as a whole aims to increase the capacity of your shoulder so that it can endure more without causing problems.

PHASE 1 of " Strengthen your shoulder": the focus is to add a considerable amount of training to the part of the shoulder, which is most affected by the condition. The exercise volume is high (many repetitions), but the peak-load is low. This makes training safer and easier to adjust .

WHAT YOU WILL EXPIRIENCE
It is OK to experience pain during exercises as long as it is bearable. There is no danger related to this. Should your symptoms flare up for more than 24h, you should adjust the elastic band as described in the chart. Resume training when the symptoms are back to normal.

IF YOU HAVE QUESTIONS?
If you have any questions to the exercise or the trial in general, please contact physiotherapist Mikkel Bek Clausen
E-mail: mikkel.bek.clausen.01@regionh.dk
Telephone: 29 42 64 01

PROS, CONS AND RISKS
At this time we do not know whether there are any advantages related to completing this program. The aim of the trial is to investigate just that. We do not expect any substantial risks related to completing this program.

This leaflet was developed by Mikkel Bek Clausen, physiotherapist and PhD-fellow at Orthopedic Department, Hvidovre Hospital. The Danish version, from which this version has been translated, was last revised 19th April 2016

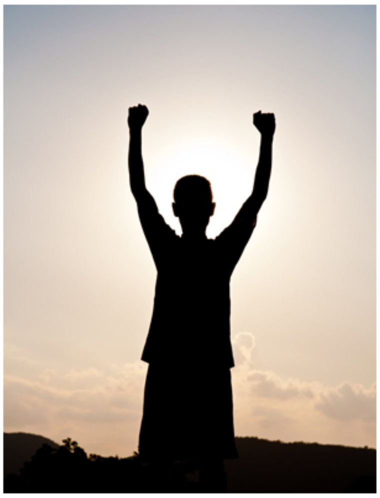

Model photo: Colourbox.com

Strengthen Your Shoulder
Program week 1 to 5

REGION Hvidovre Hospital

Exercise in Phase 1

Seated external rotation

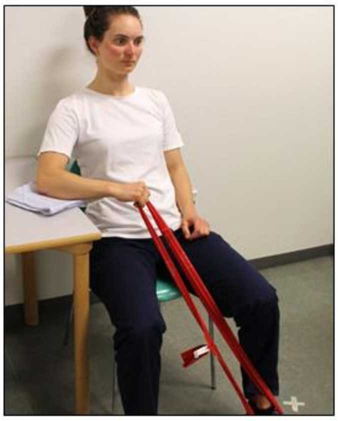

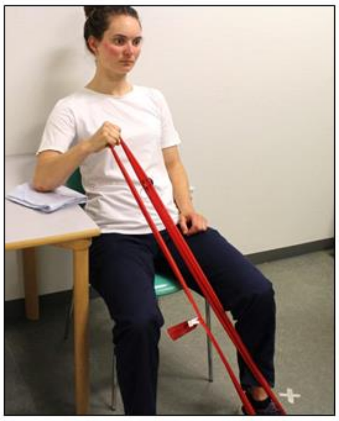

Position
- Seated with elbow supported (e.g. on a table), approx. 45° from the body
- Straight back, shoulder retracted
- Elbow in 90° flexion
- Forearm approx. horizontal

Movement
- Turn the arm so that the elastic band stretches
- Keep the elbow in 90° flexion
- Movement should only happen in the shoulder

Amount
- 3 sets, as many repetitions as possible
- Small break between sets
- Every day
- If you miss a day, do ***not*** double the amount the next day

Pace
Stretch: 2 sec
Hold: 5 sec
Release: 2 sec
Break: 2 sec

Adjusting the elastic band

When training with "Strengthen Your Shoulder" you will be adjusting the elastic band yourself.

The chart below will guide you in determining whether you should adjust the elastic band before your next training session. The elastic band is adjusted by moving the clip.

Adjustment depends on two things (see chart):
1. If your symptoms flare up for more than 24h after training
2. The number of repetitions you can do in the first set of an exercise

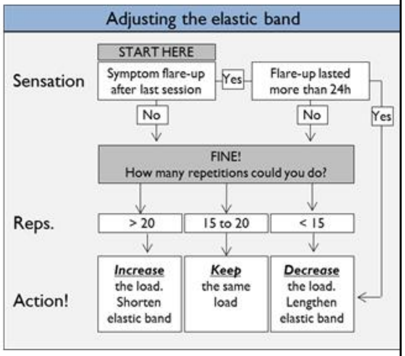

Supplement: Supplementary file 4 — Strengthen your shoulder, intervention leaflet 1. (PDF 411 kb) [file 13063_2018_2509_MOESM4_ESM.pdf]
