# Supplementary material for: The Strengthening Exercises in Shoulder Impingement trial (The SExSI-trial) investigating the effectiveness of a simple add-on shoulder strengthening exercise programme in patients with long-lasting subacromial impingement syndrome: Study protocol for a pragmatic, assessor blinded, parallel-group, randomised, controlled trial
Source: Trials. 2018 Mar 2;19:154. doi: 10.1186/s13063-018-2509-7 (PMC5833202; doi:10.1186/s13063-018-2509-7)
Supplement: Supplementary file 6 — Strengthen your shoulder, intervention leaflet 3. (PDF 588 kb) [file 13063_2018_2509_MOESM6_ESM.pdf]

Additional file 6, Strengthen your shoulder, intervention leaflet, Phase 3

THE AIM of "Strengthen your shoulder":
The program as a whole aims to increase the capacity of your shoulder so that it can endure more without causing problems.

PHASE 3 of " Strengthen your shoulder": the load is further increased to make your shoulder more robust and able to tolerate the various everyday activities that involves the shoulder. Again, one additional exercise is added to the program, this time a combined exercise targeting as many relevant muscles as possible. The exercises are now performed with a somewhat higher peak-load, but still at a level where training is safe.

WHAT YOU WILL EXPIRIENCE
It is OK to experience pain during exercises as long as it is bearable. There is no danger related to this. Should your symptoms flare up for more than 24h, you should adjust the elastic band as described in the chart. Resume training when the symptoms are back to normal.

IF YOU HAVE QUESTIONS
If you have any questions to the exercise or the trial in general, please contact physiotherapist

Mikkel Bek Clausen
E-mail: mikkel.bek.clausen.01@regionh.dk
Telephone: 29 42 64 01

PROS, CONS AND RISKS
At this time we do not know whether there are any advantages related to completing this program. The aim of the trial is to investigate just that. We do not expect any substantial risks related to completing this program.

This leaflet was developed by Mikkel Bek Clausen, physiotherapist and PhD-fellow at Orthopedic Department, Hvidovre Hospital. The Danish version, from which this version has been translated, was last revised 19th April 2016

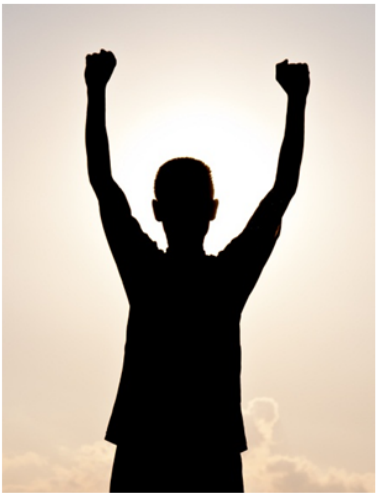

Model photo: Colourbox.com

Strengthen Your Shoulder
Program week 11 to 16

REGION Hvidovre Hospital

Exercises in Phase 3

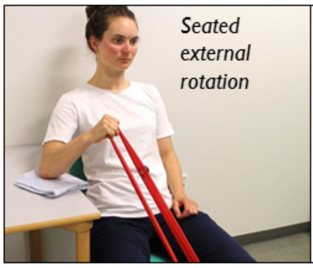

Position
- Seated with elbow supported (e.g. on a table), approx. 45° from the body
- Straight back, shoulders retracted
- Elbow in 90° flexion
- Forearm approx. horizontal

Movement
- Turn the arm, elastic band stretches
- Elbow 90° flex., only shoulder move

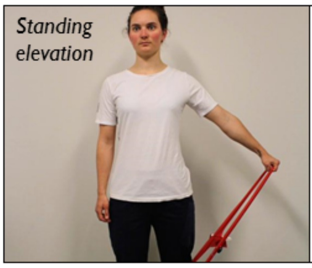

Position
- Elastic band: affected side to opp. foot
- Stand with arm stretched, the elastic band should not touch your body/leg
- Straight back, shoulders retracted

Movement
- Elevate arm sideways to 45° (half-way to horizontal)
- Arm stretched, only shoulder moves

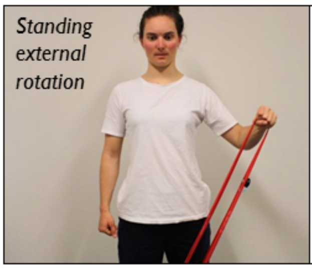

Position
- Standing with elbow elevated to approx. 45° from the body
- Straight back, shoulders retracted
- Elbow in 90° flexion
- Forearm approx. horizontal

Movement
- Turn the arm, elastic band stretches
- Elbow 90° flex., only shoulder move

Adjusting the elastic band

When training with "Strengthen Your Shoulder" you will be adjusting the elastic band yourself. The chart below will guide you in determining whether you should adjust the elastic band before your next training session. The elastic band is adjusted by moving the clip.

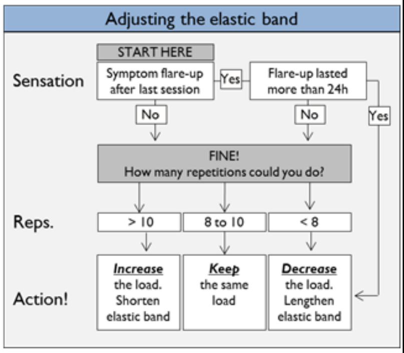

Amount and Pace

Amount
- 2 sets, as many reps as possible
- Performed ***every second day***
- If you miss a day: continue next day

Pace
- Stretch 2, hold 5, release 2, break 2
- Small break between sets
