# Supplementary material for: The Strengthening Exercises in Shoulder Impingement trial (The SExSI-trial) investigating the effectiveness of a simple add-on shoulder strengthening exercise programme in patients with long-lasting subacromial impingement syndrome: Study protocol for a pragmatic, assessor blinded, parallel-group, randomised, controlled trial
Source: Trials. 2018 Mar 2;19:154. doi: 10.1186/s13063-018-2509-7 (PMC5833202; doi:10.1186/s13063-018-2509-7)
Supplement: Supplementary file 7 — Data collection methods – study instruments. (PDF 235 kb) [file 13063_2018_2509_MOESM7_ESM.pdf]

Data collection methods for the SExSI-Trial − Study instruments

Baseline questionnaire

Using a self-developed baseline questionnaire demographic, socio-economic and disease related information, as well as expectations to outcome, are collected through standardised questionnaires with discrete answer options except for age and duration of symptoms, which are entered in years and months, respectively. All instructions to the outcome assessors are given by the primary investigator, this will ensure uniformity and quality of data.

End of treatment questionnaire

Using a self-developed questionnaire, data regarding patients' global impression of change, acceptableness of symptom state, job status and medication use are collected through standardised questionnaires with discrete answer options.

Outcome questionnaires

Shoulder pain and disability index (SPADI) consists of 13 items measuring pain (five items) and disability (eight items) [1]. Each item is scored from 0-10 (10 = worst), the pain and disability items are summed separately and, weighted equally, combined to a total score ranging from 0 to 100 (100 = worst) [2]. The psychometric properties of the Danish SPADI are well investigated in a population comparable to that included in the current study, showing good reliability (ICC 0.88) and validity [2].

Pain Catastrophizing Scale (PCS) is a patient reported questionnaire measuring pain catastrophizing, and it consists of 13 items measuring three factors; rumination (four items), magnification (three items) and helplessness (six items) [3]. Each item is scored on a 5-point Likert scale (0-4 points, 4 = worst) and combined to a total score ranging from 0 to 52. The Danish version (PCS-DAN) is found reliable (α=0.91-0.94) and valid in both a non-clinical sample and a sample of chronic pain patients [3].

EQ-5D-3L (EQ-5D) is a patient reported questionnaire measuring health related quality of life. The EQ-5D-3L consists of a descriptive system and Visual Analogue Scale of self-rated health. The descriptive system comprises five dimensions (mobility, self-care, usual activities, pain/discomfort and anxiety/depression), each containing three levels (no problem, some problems and severe problems) [4]. Scores from the descriptive part are converted to a single summary index (EQ-5D-index) reflecting a general population evaluation of the specific health state [5], using a Danish valuation set [6,7]. The self-rated health scale (EQ-5D-VAS) is scored on a vertical 20 cm visual analogue scale with "best imaginable health" and "worst imaginable health" as external anchors [4], and reflects the respondents own assessment of their health state [5]. The EQ-5D-3L questionnaire is a widely used measure of health related quality of life in scientific settings.

Maximum isometric Voluntary Contraction

Maximum isometric Voluntary Contraction (MVC) in external rotation and abduction is tested using a Hand Held Dynamometer (HHD) and a vertical bench. Subjects are asked to perform a 5 s isometric MVC against the dynamometer, during the standardized command "Go ahead-push-push-push-push and thank you" given by the examiner. Prior to each test, subjects are given standardized oral and visual presentations, and perform one successful familiarization trial. If needed, additional instructions are given. The individual test is administered two times, using the highest value as the test result. Each trial is followed by a 30 s rest period. MVC is expressed as torque in

Newton meter per kilo body weight (Nm/kg), and calculated as the test result (Newton) * lever arm (meter) * body weight $(kg)^{-1}$.

External rotation MVC test is performed with the patient sitting, with the knees and hips in 70 degrees of flexion, and both feet resting on the ground. The tested shoulder is in neutral position, and the elbow is in 90 degrees of flexion. The subject is seated close to the wall, allowing the dynamometer to be held between the wall and the distal aspect of the forearm. The dynamometer is placed dorsally on the forearm, with the distal aspect of the dynamometer's sensor aligned just proximal to the radiocarpal joint. The subject exerts maximum effort against the dynamometer. The examiner is positioned in front of the subject, to ensure force is only generated through external shoulder rotation. The lever arm length is measured from the centre of the dynamometer's sensor to the lateral epicondyle of the humerus. The reliability of the test is found high (ICC 0.91) [8].

Abduction MVC test is performed with the patient sitting, with the knees and hips in 70 degrees of flexion, and both feet resting on the ground. The tested shoulder is in neutral position, and the elbow is extended to 0 degrees. The subject is seated close to the wall, allowing the dynamometer to be held between the wall and the distal aspect of the forearm. The dynamometer is placed dorsally on the forearm, with the distal aspect of the dynamometers' sensor aligned just proximal to the radiocarpal joint. The subject exerts maximum effort against the dynamometer. The examiner is positioned in front of the subject, to ensure force is only generated through shoulder abduction. The lever arm length is measured from the centre of the dynamometer's sensor to the lateral aspect of the acromion. The reliability of the test is found high (ICC 0.93) [8].

Active Range of Movement in abduction

The subject is standing, with the arm in anatomical position, and the elbow extended to 0 degrees. The inclinometer is reset on a vertical surface and the subject raises the arm in the coronal plane, towards the ceiling with the examiner positioned behind the subject, ensuring the absence of compensatory movements of the torso. A measurement is taken with the inclinometer aligned parallel to the humerus, in close proximity to the insertion of the deltoid muscle. The reliability of the test is good (ICC 0.95) [9].

Cuff algometry (computer-controlled)

Cuff Pressure Pain Detection threshold (PDT) and cuff Pressure Pain Tolerance Threshold (PTT) will be used to measure Temporal Summation of Pain (TSP) and Conditioned Pain Modulation (CPM). PDT and PTT will be assessed by a computer-controlled cuff pressure algometer (Nocitech, Denmark and Aalborg University, Denmark). Computer controlled cuff algometry have previously been widely used to study central pain mechanisms [10–12]. A 13-cm wide silicone tourniquet cuff (VBM, Germany) with an equal-sized proximal and distal chamber will be wrapped around the lower leg on the same side as the shoulder pain. The leg is used in order to examine central pain mechanisms independently from local pain. The cuff pressure will increase with a rate of 1 kPa/s simultaneously in both chambers and the maximal pressure limit is 100 kPa. The participants use an electronic visual analogue scale (VAS) to rate their pressure-induced pain intensity and a button to release the pressure. The electronic VAS will be sampled at 10 Hz. Zero and ten cm extremes on the VAS are defined as "no pain" and "maximal pain", respectively. The participants will be instructed to rate the pain intensity continuously on the electronic VAS from the first sensation of pain and to press the pressure release button when the pain is intolerable. The pressure value when the subject rates the sensation of pain as 1 cm on the VAS is defined as the Pain Detection Threshold (PDT) and the pressure recorded when the subject terminated the cuff inflation is defined as the Pressure Pain Tolerance Threshold (PTT) [10]. A template regarding the information given to participants prior to the commencement of Cuff

Algometry tests is used in order to ensure that sufficient and similar instructions and information are given to all participants. Measures of PDT and PTT using computerized cuff algometri are found reliable (ICC 0.7 to 0.9) [13].

Temporal Summation of Pain (TSP) is quantified as the increase in pain recorded on an electronic VAS scale (range: 0-10 cm), during repeated standardized pressure induced pain stimuli. TSP is assessed by the computer controlled cuff algometer (NociTech, Denmark). Ten 1-second cuff pressure stimuli with 2-second inter stimulus interval are delivered to the lower leg by simultaneous inflation of both cuff chambers at an intensity equivalent to the recorded PTT. During the between stimulus interval, a constant non-painful pressure of 5 kPa is maintained to ensure that the cuff does not move. Participants are instructed to rate the pain intensity continuously on the electronic VAS. For each stimulus, the VAS score at 1 s following the stimuli is extracted. TSP is quantified as the difference between a mean of the VAS scores for the last three stimulations and the mean of the VAS scores for the first four stimulations [13].

Conditioned Pain Modulation (CPM) is quantified as the increase in PDT and PTT (test stimulus), when experimental tonic pain is induced in the contralateral lower leg (conditioning stimulus), using a 13-cm-wide tourniquet cuff (VBM, Düsseldorf, Germany) for both stimuli. For the conditioning stimulus, the computer-controlled cuff algometer maintain a constant pressure corresponding to 75% of the PTT pressure from an initial test of PTT [14], while a new measurement of PDT and PTT (test stimulus) is conducted in the lower leg on the same side as the shoulder pain.

Other pain variables

Pressure Pain Threshold is measured using a hand-held pressure algometer (Somedic Sales AB, Sweden) with a stimulation area of 1 cm^2 placed perpendicular to the skin. Pressure is applied at a rate of 30 kPa/s and the subjects are instructed to indicate when the sensation changes from a sensation of pressure to the first sensation of pain. Measurements are done with the individuals seated upright with the unaffected shoulder towards and touching the wall for support during testing. PPT is assessed at the following sites: 1) the muscle belly of the deltoid muscle, between the deltoid tuberosity and the anterolateral aspect of the acromion, 2) the middle point of the supraspinatus muscle belly, over the fossa of the scapula[15], 3) the middle of the infraspinatus muscle belly[15] and 4) at the site of worst pain as described by the participant. PPT is measured twice at each site, with the average of measurements calculated and used for analyses. Similar measures of PPT in the arm have demonstrated good reliability (ICC 0.87) [13]. In order to ensure uniformity and quality of data, multiple instruction sessions were performed with each outcome assessors, supervised by the primary investigator.

Pain last week is measured on a numeric pain rating scale (NPRS, 0 to 10, 0 = no pain), using standardised verbal anchors for the instruction [16]. Participants are asked to report their current pain and their least, worst and average pain during the last week on a scale from 0 to 10, 0 being no pain and 10 being the worst imaginable pain. For analyses, the mean of least and average pain is used, as this is the most valid composite score [16].

Pain during tests of MVC and abduction ROM is measured on a numeric pain rating scale (NPRS, 0 to 10, 0 = no pain), using standardised verbal anchors for the instruction [16]. Immediately after each test, the participant answer the standardized question “On a scale from 0 to 10, 0 being no pain and 10 being the worst imaginable pain, how much did it hurt to do the test?” and the number is recorded in the Case Report Form.

Clinical tests

Scapula Dysfunction (rated positive/negative) is measured using the modified Scapula Assistance Test (mSAT) as described by Rabin et al. [17]. First, the participant is asked to elevate the arm as

high as possible in a flexion movement, and afterwards to rate the pain felt during the movement on an 11-point NPRS scale. Secondly, the same procedure is performed, but with the assessor facilitating upward rotation of the scapula. A reduction of two or more points on the NPRS scale is considered a positive test result. The mSAT has demonstrated acceptable reliability (Kappa 0.6) in impingement patients [17,18].

Scapula Dyskinesia (rated yes/no) is measured using the Scapula Dyskinesia Test (SDT) as described by McClure et al. [19]. Participants are asked to simultaneously elevate their arms as high as possible, in flexion and abduction respectively, to perform five 3-seconds repetitions of bilateral weighted shoulder elevation in each direction. For each repetition, the affected shoulder is judged as normal, subtle dyskinesia or obvious dyskinesia. Flexion and abduction is then, separately, judged as normal (≥3 normal repetitions), obvious dyskinesia (obvious dysrhythmia or winging in ≥3 repetitions), or subtle dyskinesia (minor, questionable or obvious dysrhythmia or winging in ≥2 repetitions and not judged as obvious). The overall ratings are: 1) obvious, if either flexion or abduction is judged as obvious dyskinesia; 2) subtle, if both flexion and abduction is judged as subtle dyskinesia; or 3) normal, in all other cases. Weights are chosen based on body weight, 1.4 kg for participants weighing ≤68 kg and 2.3 kg for those weighing >68 kg. The SDT has demonstrated acceptable reliability [19] and validity [20] in overhead athletes. For the purpose of further analyses all ratings were further dichotomized to 'yes' (ratings of obvious dyskinesia) and 'no' (ratings of normal or subtle dyskinesia). For the study described in this protocol, a standard modification procedure was used, as we expected a significant proportion of patients would not be able to elevate the arm sufficiently with the prescribed weight to allow judgement regarding the presence of dyskinesia. Accordingly, when a patient was unable to elevate the arm above 90 degrees with the prescribed weight during the first repetition, the weight was regressed one step (from 2.3 kg to 1.4 kg to unweighted) until the patient was able to do so. In cases when a patient was unable to elevate the arm to at least 60 degrees, as visually judged by the assessor, in one or more of the following repetitions, the weight was regressed and the test redone. The inter-observer reliability of the modified SDT procedure, as is applied in the current project, has been tested in relation to the conduction of this study, with two outcome assessors rating the same patient at the same time during a follow-up testing session, and each outcome assessor blinded to the rating of the other. This has been done in a convenience sample of 25 enrolled patients and revealed a kappa value of 0.77 (95%CI 0.52 to 1.0) for the dichotomized rating of scapula dyskinesia.

References

1. Roach KE, Budiman-Mak E, Songsiridej N, Lertratanakul Y. Development of a shoulder pain and disability index. Arthritis Care Res Off J Arthritis Health Prof Assoc. 1991;4:143–9.

2. Christiansen DH, Andersen JH, Haahr JP. Cross-cultural adaption and measurement properties of the Danish version of the Shoulder Pain and Disability Index. Clin Rehabil. 2013;27:355–60.

3. Kjøgx H, Zachariae R, Pfeiffer-Jensen M, Kasch H, Svensson P, Jensen TS, et al. Pain frequency moderates the relationship between pain catastrophizing and pain. Front Psychol. 2014;5:1421.

4. Szende A, Williams A, EuroQol Group. Measuring self-reported population health: an international perspective based on EQ-5D. [Budapest] Hungary: SpringMed Publishing; 2004.

5. EuroQol - EQ-5D-3L [Internet]. [cited 2016 Jan 14]. Available from: http://www.euroqol.org/faqs/eq-5d-3l.html

6. Szende A, Oppe M, Devlin NJ, EuroQol Group, editors. EQ-5D value sets: inventory, comparative review, and user guide. Dordrecht: Springer; 2007.

7. Sørensen J, Davidsen M, Gudex C, Pedersen KM, Brønnum-Hansen H. Danish EQ-5D population norms. Scand J Public Health. 2009;37:467–74.

8. Clausen MB, Witten A, Holm K, Christensen KB, Attrup ML, Hölmich P, et al. Glenohumeral and scapulothoracic strength impairments exists in patients with subacromial impingement, but these are not

reflected in the shoulder pain and disability index. BMC Musculoskelet Disord [Internet]. 2017 [cited 2017 Jul 18];18. Available from: http://bmcmusculoskeletdisord.biomedcentral.com/articles/10.1186/s12891-017-1667-1

9. Kolber MJ, Vega F, Widmayer K, Cheng M-SS. The reliability and minimal detectable change of shoulder mobility measurements using a digital inclinometer. Physiother Theory Pract. 2011;27:176–84.

10. Graven-Nielsen T, Wodehouse T, Langford RM, Arendt-Nielsen L, Kidd BL. Normalization of widespread hyperesthesia and facilitated spatial summation of deep-tissue pain in knee osteoarthritis patients after knee replacement. Arthritis Rheum. 2012;64:2907–16.

11. Skou ST, Graven-Nielsen T, Rasmussen S, Simonsen OH, Laursen MB, Arendt-Nielsen L. Widespread sensitization in patients with chronic pain after revision total knee arthroplasty. Pain. 2013;154:1588–94.

12. Rathleff MS, Petersen KK, Arendt-Nielsen L, Thorborg K, Graven-Nielsen T. Impaired Conditioned Pain Modulation in Young Female Adults with Long-Standing Patellofemoral Pain: A Single Blinded Cross-Sectional Study. Pain Med [Internet]. 2015; Available from: http://painmedicine.oxfordjournals.org/content/early/2015/12/12/pm.pnv017.abstract

13. Graven-Nielsen T, Vaegter HB, Finocchietti S, Handberg G, Arendt-Nielsen L. Assessment of musculoskeletal pain sensitivity and temporal summation by cuff pressure algometry: a reliability study. Pain. 2015;156:2193–202.

14. Graven-Nielsen T, Izumi M, Petersen KK, Arendt-Nielsen L. User-independent assessment of conditioning pain modulation by cuff pressure algometry. Eur J Pain Lond Engl. 2017;21:552–61.

15. Hidalgo-Lozano A, Fernández-de-las-Peñas C, Alonso-Blanco C, Ge H-Y, Arendt-Nielsen L, Arroyo-Morales M. Muscle trigger points and pressure pain hyperalgesia in the shoulder muscles in patients with unilateral shoulder impingement: a blinded, controlled study. Exp Brain Res. 2010;202:915–25.

16. Jensen MP, Turner JA, Romano JM, Fisher LD. Comparative reliability and validity of chronic pain intensity measures. Pain. 1999;83:157–62.

17. Rabin A, Irrgang JJ, Fitzgerald GK, Eubanks A. The intertester reliability of the Scapular Assistance Test. J Orthop Sports Phys Ther. 2006;36:653–60.

18. Struyf F, Nijs J, Mottram S, Roussel NA, Cools AMJ, Meeusen R. Clinical assessment of the scapula: a review of the literature. Br J Sports Med. 2012;

19. McClure P, Tate AR, Kareha S, Irwin D, Zlupko E. A Clinical Method for Identifying Scapular Dyskinesis, Part 1: Reliability. J Athl Train. 2009;44:160–4.

20. Tate AR, McClure P, Kareha S, Irwin D, Barbe MF. A Clinical Method for Identifying Scapular Dyskinesis, Part 2: Validity. J Athl Train. 2009;44:165–73.

This is an Additional File for the article titled:

The Strengthening Exercises in Shoulder Impingement trial (The SExSI-trial) investigating the effectiveness of a simple add-on shoulder strengthening exercise programme in patients with long lasting subacromial impingement syndrome: Study protocol for a pragmatic, assessor blinded, parallel-group, randomised, controlled trial
